# Neuropathology in chronic traumatic encephalopathy: a systematic review of comparative post-mortem histology literature

**DOI:** 10.1186/s40478-022-01413-9

**Published:** 2022-08-06

**Authors:** Helen C. Murray, Chelsie Osterman, Paige Bell, Luca Vinnell, Maurice A. Curtis

**Affiliations:** Department of Anatomy and Medical Imaging and Centre for Brain Research, Faculty of Medical and Health Science, University of Auckland, Auckland, 1023 New Zealand

**Keywords:** Chronic traumatic encephalopathy, Dementia pugilistica, Neuropathology, Systematic review

## Abstract

Chronic traumatic encephalopathy (CTE) is a neurodegenerative disease associated with repetitive head trauma and is characterised by the perivascular accumulation of hyperphosphorylated tau (p-tau) in the depths of cortical sulci. CTE can only be diagnosed postmortem and the cellular mechanisms of disease causation remain to be elucidated. Understanding the full scope of the pathological changes currently identified in CTE is necessary to identify areas requiring further research. This systematic review summarises the current literature on CTE pathology from postmortem human tissue histology studies published until 31 December 2021. Publications were included if they quantitively or qualitatively compared postmortem human tissue pathology in CTE to neuropathologically normal cases or other neurodegenerative diseases such as Alzheimer’s disease (AD). Pathological entities investigated included p-tau, beta-amyloid, TDP-43, Lewy bodies, astrogliosis, microgliosis, axonopathy, vascular dysfunction, and cell stress. Of these pathologies, p-tau was the most frequently investigated, with limited reports on other pathological features such as vascular dysfunction, astrogliosis, and microgliosis. Consistent increases in p-tau, TDP-43, microgliosis, axonopathy, and cell stress were reported in CTE cases compared to neuropathologically normal cases. However, there was no clear consensus on how these pathologies compared to AD. The CTE cases used for these studies were predominantly from the VA-BU-CLF brain bank, with American football and boxing as the most frequent sources of repetitive head injury exposure. Overall, this systematic review highlights gaps in the literature and proposes three priorities for future research including: 1. The need for studies of CTE cases with more diverse head injury exposure profiles to understand the consistency of pathology changes between different populations. 2. The need for more studies that compare CTE with normal ageing and AD to further clarify the pathological signature of CTE for diagnostic purposes and to understand the disease process. 3. Further research on non-aggregate pathologies in CTE, such as vascular dysfunction and neuroinflammation. These are some of the least investigated features of CTE pathology despite being implicated in the acute phase response following traumatic head injury.

**Supplementary Information:**

The online version contains supplementary material available at 10.1186/s40478-022-01413-9.

## Introduction

Chronic traumatic encephalopathy (CTE) is a neurodegenerative disease associated with repetitive head injury. CTE neuropathology can only be confirmed by postmortem assessment and has been identified in athletes from a range of sports, including American football, soccer, rugby, ice hockey, lacrosse, mixed martial arts, wrestling and boxing at amateur and professional levels, as well as military veterans, victims of domestic violence, individuals who had multiple falls and individuals who suffered from head-banging behaviour [60, 62]. Despite the prevalence of repetitive head injury in modern society, our understanding of CTE and the underlying neuropathology has evolved remarkably slowly over the last century.

The earliest reports linking repetitive head injury in boxers with distinct neuropathology led to the adoption of the terms ‘dementia pugilistica’ or ‘punch drunk syndrome’ [29, 56]. However, within the last 2 decades, an abundance of reports have demonstrated that the pathology of dementia pugilistica is not unique to boxers et al. [31, 52, 60, 62, 70–72, 77]. This acknowledgement that a broad range of head injury exposure sources are associated with neurodegeneration fuelled the adoption of the term ‘chronic traumatic encephalopathy’ in modern literature. It is widely considered that these two terms describe equivalent neuropathology and the term ‘dementia pugilistica’ is therefore historically relevant to any literature survey of CTE neuropathology et al. [20, 39, 57].

Since the first report of CTE neuropathology in a cohort of 15 former boxers by Corsellis et al. in 1973 et al. [29] there has been a need to define the distinguishing features of CTE pathology for postmortem assessment. The McKee criteria, first presented in 2013, proposed that the pathological diagnosis of CTE required the presence of focal perivascular accumulations of p-tau within neurons as neurofibrillary tangles (NFTs) and neuropil threads and within astrocytes as astrocytic tangles, specifically with a predilection for the depths of cortical sulci et al. [60]. A consensus group refined the definition of this pathognomonic lesion in 2015 with details of supporting neuropathological features [58]. These features included p-tau accumulations distributed in the superficial layers of the cortex and within the CA1 and CA4 subregions of the hippocampus, with dendritic swellings also present in CA4. In addition, p-tau immunoreactive astrocytes were deemed supporting features and non-diagnostic in isolation given the increasing recognition of age-related tau astrogliopathy (ARTAG). Other non-p-tau related pathologies were also detailed such as macroscopic indications of previous head trauma and the presence of TDP-43 accumulations in the hippocampus, anteromedial temporal cortex, and amygdala.

The findings from a second consensus meeting published in 2021 further clarified the definition of the pathognomonic lesion to emphasise that the p-tau should include NFTs, with or without astrocytes, in the deep layers of the cortical sulci and should not be limited to the subpial and superficial layers. Presence of a p-tau lesion was defined as the minimum threshold criteria for CTE diagnosis. The staging of pathological severity was also refined as ‘low CTE’ or ‘high CTE’ based on the presence of NFTs in supporting regions [16]. For a detailed description and visualisation of CTE pathological features see reference [57]. These diagnostic criteria allow for more standardised classification of CTE pathology, but it is widely acknowledged that multiple neuropathologies such as CTE p-tau lesions, beta-amyloid plaques, cerebral amyloid angiopathy, Lewy body disease, and TDP-43 proteinopathy can be present in the brain of a single individual, and that this cooccurrence is more common with increasing age [83].

Brain banking and neuropathological research efforts have led to the development of these core features that can be systematically and routinely identified in CTE cases, largely centred around the presence and location of p-tau. However, non-tau-related pathological changes such as microvascular alterations, white matter damage, reactive microgliosis and astrogliosis, are still vastly understudied. The detailed characterisation of these pathologies and their distribution relative to CTE p-tau lesions would accelerate our understanding of the disease process and could help distinguish CTE from other diseases and normal aging.

CTE neuropathology research is at the edge of a new era. With intense public interest in CTE due to the potential for improving athlete safety and neurological disease prevention, it is important to understand this pathology. Additionally, with a more global network of systematic brain tissue collection, and emerging high-content technologies for studying anatomy, there is an opportunity to accelerate research that will explain the processes linking repetitive head injury and neurodegeneration. We conducted a systematic review of CTE human tissue histology studies to better understand the scope of the pathological changes currently identified in CTE and identify the most promising areas for future research efforts. Here, we reviewed and summarised the studies that assessed CTE brain tissue alongside normal aging or other neurodegenerative diseases. Our findings summarise the current landscape of comparative CTE literature and highlight the pathological features that warrant more detailed investigation.

## Methods

### Systematic search and summary of literature

**Fig. 1:**
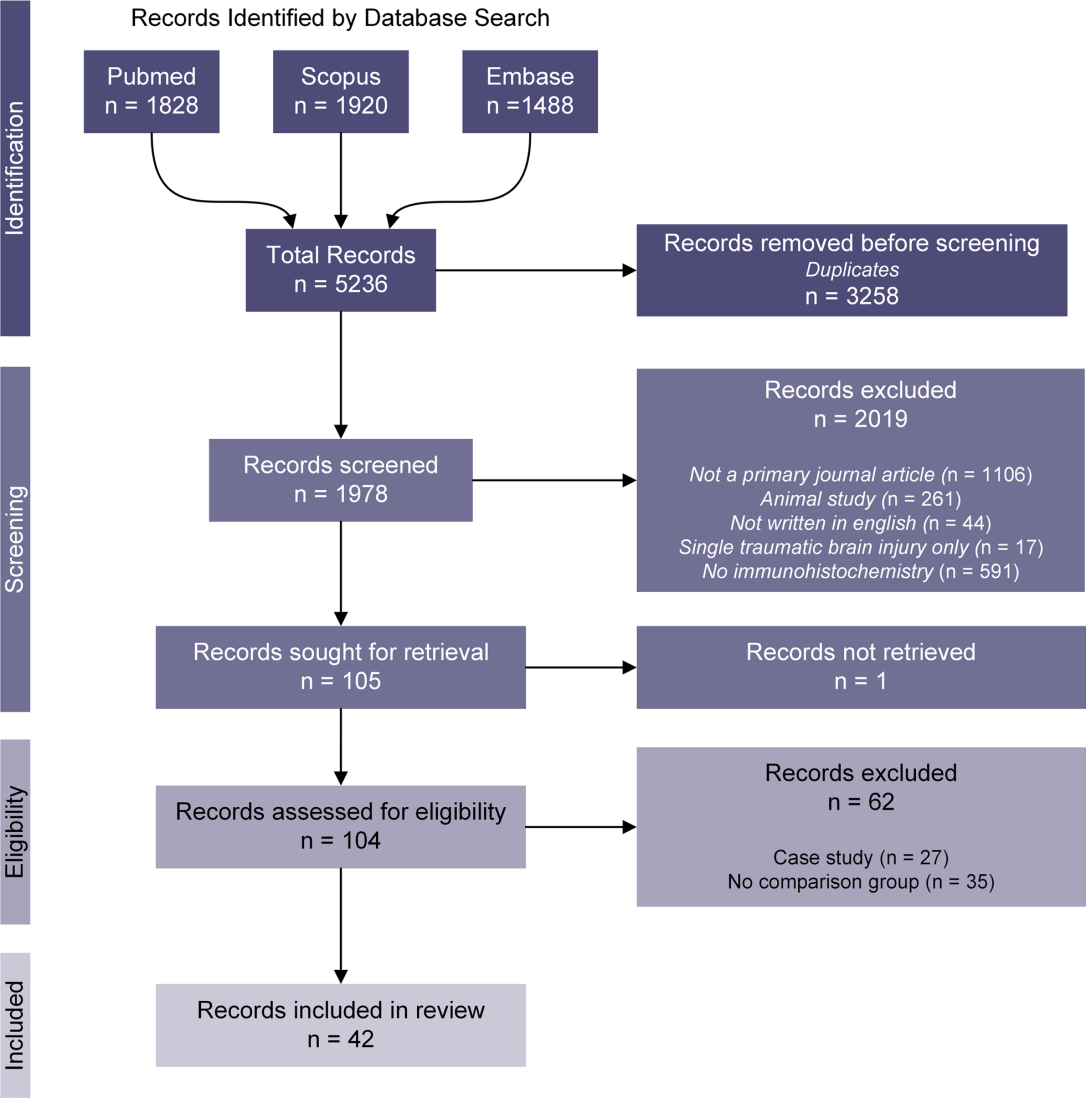
PRISMA flow diagram for selection of records included in this review. The search terms “chronic traumatic encephalopathy”, “dementia pugilistica”, “repetitive mild traumatic brain injury”, and “chronic traumatic encephalopathy neuropathology” were used to query the Pubmed, Scopus, and Embase databases for all relevant articles published until 31 December 2021, resulting in 5236 records. Duplicate records (3258) were removed using an excel query. The remaining 1978 records were screened to determine only those that examined human postmortem tissue. From these 104 records, all case studies (only one CTE case described) and descriptive studies (no comparison to normal aging or other disease groups) were excluded, resulting in 42 articles that met the eligibility criteria for this review

A systematic search for relevant articles published until 31 December 2021 was conducted using Pubmed, Scopus and Embase databases. The terms “chronic traumatic encephalopathy”, “dementia pugilistica”, “repetitive mild traumatic brain injury”, and “chronic traumatic encephalopathy neuropathology” were queried in each database and returned 5236 records in total. The process for record selection followed the Preferred Reporting Items for Systematic Reviews and Meta-Analyses (PRISMA) guidelines, as illustrated in Fig. 1. Duplicate records were first removed using an excel query resulting in 1978 records. The title and abstract of these records were manually screened to identify publications that included an investigation of human postmortem tissue histology from cases defined by the authors as CTE or dementia pugilistica. The full text of the 104 records that fit these criteria was retrieved and manually screened to identify publications where postmortem tissue histology from more than one CTE case was compared to either a neuropathologically normal case group, an Alzheimer’s disease case group or another neurodegenerative disease case group. These publications are referred to from here on as comparative studies and we refer to the neuropathologically normal case group as ‘normal aging’. Publications that examined only one CTE or dementia pugilistica case were labelled as case studies and excluded. Publications that only examined CTE or dementia pugilistica cases and did not compare (qualitatively or quantitatively) to another case group were labelled as descriptive and excluded. This protocol resulted in 42 publications that met the eligibility criteria for this review. Due to the low number of publications that met the review criteria, no study quality threshold was applied. Instead, we have noted in the results where a conclusion is derived from a publication with a small number of CTE cases.

Data from each publication was extracted and tabulated (supplementary data). Items were listed as ‘Not Reported’ if the information was not found in the article, supplementary information, or any referenced articles in the methods section. Items were listed as ‘Not applicable’ if they were not relevant to the study.

### Objectives

This review aimed to summarise the current histology literature on the neuropathological changes in CTE relative to normal aging and Alzheimer’s disease from human postmortem tissue studies. In doing so, we aimed to identify opportunities for future neuroanatomical studies. To achieve these aims, we sought to address two key objectives:

1. Determine the source and demographics of the CTE cases that have been examined in comparative postmortem tissue histology studies
2. Summarise the pathological changes that have been compared between CTE, normal aging and Alzheimer’s disease cases using histology techniques.

### Classification of brain bank that contributed the CTE cases

To assess the number of studies using CTE tissue from different brain banks, the methods section of each of the 42 publications included in this review was examined to determine the brain bank(s) that provided the CTE tissue for the study. Publications that state the CTE cases were provided by the Boston University Chronic Traumatic Encephalopathy Centre or the Understanding Neurological Injury and Traumatic Encephalopathy (UNITE) study were counted toward the total of the Veteran Affairs-Boston University-Concussion Legacy Foundation (VA-BU-CLF) brain bank. The Boston University Alzheimer’s Disease Centre and Framingham Heart Study were considered separate brain banks despite being based at Boston University. Publications that referred to the ‘Runwell Hospital’ or ‘Corsellis Collection’ were considered the same brain bank based on the publication by Corsellis et al. [29]. Similarly, studies conducted on tissue acquired by Dr Omalu were grouped as the ‘Omalu Collection’. It is important to note that we were not able to determine whether specific CTE cases were used for multiple studies as unique case identification was not provided by most publications.

### Classification of pathology category and direction of pathological change

Publications were classified into a pathology category based on the antibodies/stains performed in the study. To be included in a pathology category, the study must examine a relevant antibody/stain in more than one CTE case and compare the result qualitatively, semi-quantitatively, or quantitatively with a normal aging group and/or another neurodegenerative disease group. Publications were included in multiple pathology categories if these conditions were met. Where available and appropriate, the difference in pathology between CTE and other case groups was summarised as either increased, no change, or decreased. The method of comparison and the brain regions compared varied between studies, so this summary was only made where a qualitative description, a semi-quantitative description, or a quantitative measurement of the difference between two groups for a defined brain region was provided. The measurement, method of quantification and regions compared are summarised for each study in the Additional file 1. Publications were not included in this pathology results summary if the comparison between groups was complex and could not be accurately summarised (such as a comparison of pathology distribution in different cortical layers).

## Results

### Summary of publications and demographics of CTE cases

Our systematic review identified 104 postmortem human tissue studies of CTE, the first of which was by Corsellis et al. in 1973 [29]. Of these studies, 42 compared CTE tissue to that of neuropathologically normal aged individuals or another disease group. Prior to 2000, only nine comparative studies of CTE postmortem tissue examined the brains of former boxers with CTE, referred to as dementia pugilistica.

**Fig. 2:**
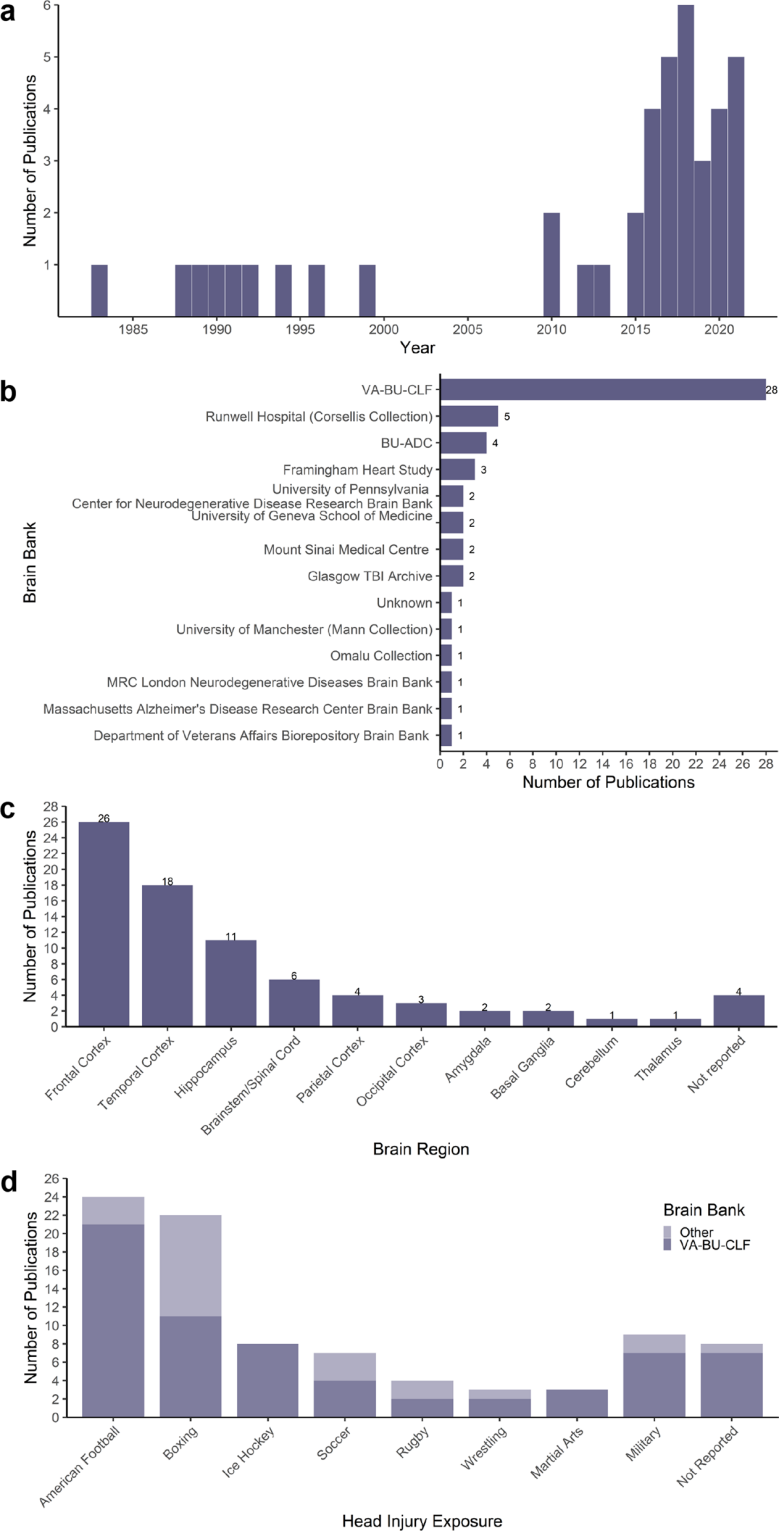
Characteristics of the systematic review results. **a** Histogram of the number of comparative CTE neuropathology studies published by year. **b** Bar graph illustrating the number of publications using CTE postmortem tissue from each brain bank. **c** Bar graph of the number of publications investigating different brain regions. **d** Stacked bar graph of the number of publications from the VA-BU-CLF brain bank or other brain banks that study cases from each head injury exposure category

By plotting the number of publications by year we found that the frequency of comparative CTE postmortem tissue studies has increased since 2015 (Fig. 2a). This coincides with consensus confirmation of the CTE pathognomonic lesion and publication of the NINDS pathological criteria [58]. It further correlates with the increase in postmortem brain donations to the VA-BU-CLF brain bank due to increased efforts to promote brain donation by the CLF, including the relaunch of the brain donation campaign and a transition to an online signup system [86]. Assessment of the 42 tissue studies indicated that the CTE cases were sourced from at least 13 different brain banks. Nine studies sourced CTE tissue from more than one brain bank. We also identified that 66.7% of the studies use tissue from the VA-BU-CLF brain bank, which has the most extensive brain collection from individuals with significant head injury exposure (Fig. 2b).

Our assessment of the CTE comparative studies showed that a wide range of brain regions have been investigated. The frontal cortex is the most frequently studied region and is also the most common site of CTE lesions [82]. The temporal cortex and hippocampus were also frequently studied and are regions where p-tau pathology appears in the later, more severe stages of CTE [60] (Fig. 2c).

Our analysis also showed that contact sport was the most frequent head injury exposure in the CTE studies. Postmortem tissue from former athletes representing a range of sports has been examined, including American football, boxing, ice hockey, soccer, rugby, wrestling, and martial arts. American football athletes were the most frequently studied head injury exposure population (25/42 publications), and 84% of those studies obtained the tissue from the VA-BU-CLF brain bank. Boxing was the second most frequently studied form of head injury exposure (22/42 publications), with 50% of these cases from the VA-BU-CLF brain bank. This reflects the studies prior to 2000 that primarily examined the brains of former boxers as the historical classification of dementia pugilistica or ‘punch drunk syndrome’ was considered specific to this sport [39] (Fig. 2d). Military service was the most frequent non-sport-related head injury exposure (9/42 publications), with 78% of cases obtained from the VA-BU-CLF brain bank.

Of the 31 publications that reported the sex of the CTE cases in their study, all cases were male.

### Pathological changes in CTE

After reviewing the 42 publications, we assigned them to one of nine pathology categories based on the antibody labelling or stains performed in the study, summarised in Table 1. Many studies included an assessment of multiple pathologies, and therefore a study could be assigned to more than one group (summarised in Additional file 1). The findings for each comparison between CTE cases and other case groups were summarised for each pathology category.

**Table 1:** Summary of papers by pathology category

Category	Number of publications	Number of CTE cases (n)	Protein (antibodies) investigated
Tau	31	Adams et al. [2], n = 86 Arena et al. [6], n = 22 Arena et al. [7], n = 14 Armstrong et al. [10], n = 11 Armstrong et al. [11], n = 8 Armstrong et al. [12], n = 11 Armstrong et al. [13], n = 4 Cherry et al. [26], n = 48 Cherry et al. [27], n = 45 Cherry et al. [23], n = 41 Combs and Kanaan [28] n = 5 Dale et al. 30], n = 11 Geddes et al. [36], n = 4 Goldstein et al. [37], n = 8 Hof et al. [40], n = 3 Hsu et al. [44], n = 14 Kanaan et al. [46], n = 9 Kaufman et al. 48], n = 27 Kondo et al. [50], n = 16 Lucke-Wold et al. [54], n = 2 Mann et al. [55], n = 4 McKee et al. [59], n = 9 McKee et al. [60], n = 68 McKenzie et al. [61], n = 5 Moszczynski et al. [66], n = 5 Puvenna et al. [74], n = 6 Roberts et al. [75], n = 8 Roberts et al. [76] n = 20 Seo et al. [78], n = 6 Standring et al. [80], n = 251 Walt et al. [88], n = 9	Bielschowski stain [30, 59, 76] Phospho-neurofilament medium (BF10 [30, 75]) Tau 210 kDa (RT97 [75]) Pan-tau (aa210-230) (TAU-5 [74]) Conformation dependent p-tau (Alz-50 [61], GT7 [7], GT38 [6, 7]) Tau C-terminus (A0024 [36]) PAD exposed p-tau (TNT1 [46], TNT2 [28]) oligomeric p-tau (TOC1 [46], T22 [66]) tau cleaved D421 (TauC3 [46]) Tau N-terminus (Tau13 [7, 28]) 3R p-tau [7, 23] 4R p-tau [7, 23] PPP3CA [78] Phospho-tau pS199 [78] Phospho-tau pS202/pT205 (AT8 [10–13, 23, 26, 27, 44, 48, 59, 60, 74, 78], CP-13 [6, 7, 74]) Phospho-tau pS212/pT214 (AT100 [7]) Phospho-tau pS262 [7] Phospho-tau pS396/pS404 (PHF-1 [7, 59]) Phospho-tau pS396 [78] Phospho-tau pS422 [46] Phospho-tau pT175 [66] Phospho-tau pT231 [66]
Beta-amyloid	6	Armstrong [8], n = 6 Armstrong [9], n = 6 Hof et al. [40], n = 3 Roberts et al. [76], n = 20 Standring et al. [80], n = 251 Stein et al. [84], n = 114	Beta-Amyloid 1–42 (4G8 [40, 80], 4D12 [76], AB5078P [8–10, 12, 84]) Beta-Amyloid 1–40 (AB5074P [84]) Thioflavin S stain [40]
TDP-43	4	Anderson et al. [5], n = 18 King et al. [49], n = 3 McKee et al. [59], n = 9 McKee et al. [60] n = 68	Pan TDP-43 [49, 59, 60] Phospho-TDP-43 [5, 49]
Lewy body	4	Adams et al. [2], n = 86 Kaufman et al. [48], n = 27 Mann et al. [55], n = 4 McKee et al. [60], n = 68	Alpha-synuclein [2, 48, 55, 60]
Astrogliosis	4	Chancellor et al. [21], n = 8 Cherry et al. [26], n = 48 Cherry et al. [27], n = 45 Hsu et al. [44], n = 14	GFAP [21, 26, 44] CD44 [21] NQO1 [27]
Microgliosis	3	Cherry et al. [25] n = 46 Cherry et al. [26], n = 48 Goldstein et al. [37], n = 8	HLA-DR [37] Iba1 [25, 26] CD68 [26] TMEM119 [25]
Axonopathy	5	Bi et al. [15], n = 5 Chancellor et al. [21], n = 8 Goldstein et al. [37], n = 8 McKee et al. [60], n = 68 Warling et al. [89], n = 11	Phospho-neurofilament heavy [37, 60] βIII tubulin [15] Myelin-associated glycoprotein [15] Olig2 [21] Golgi stain [89]
Vascular dysfunction	3	Adams and Bruton [1], n = 18 Buée et al. [19], n = 2 Standring et al. [80], n = 251	Beta-amyloid [80] Perivascular iron deposition [1] Heparan sulfate proteoglycan [19]
Cell stress	3	Anderson et al. n = 18 Dale et al. [30] n = 11 Lucke-Wold et al. [54], n = 2	XBP1 [54] phospho-eIF2a [54] ATF6 [54] CHOP [54] Ubiquitin [30] NUP62 [5]

We identified 29/42 publications comparing CTE cases with neuropathologically normal aged cases with no disease-associated neuropathology (from here on referred to as normal aged cases). 19/42 publications compared CTE and AD cases and 7/42 compared CTE cases with both normal aging and AD groups. 17/42 publications compared CTE to another disease group: traumatic brain injury [1], repetitive head injury without dementia [26], Down’s syndrome [8, 9, 19, 55, 61], Parkinson’s disease [55], progressive supranuclear palsy [7, 28, 49, 55], amyotrophic lateral sclerosis (ALS) [55, 59, 66, 88], Picks disease [7, 19, 28], Guam dementia [19], Dementia with Lewy bodies [2, 8, 9, 49], Corticobasal degeneration [7–9, 28, 49], multiple system atrophy [49], Huntington’s disease [49], epilepsy [74], frontotemporal dementia (FTD) [44, 48], primary age-related tauopathy (PART) [48] and aging-related tau astrogliopathy (ARTAG) [7].

**Fig. 3:**
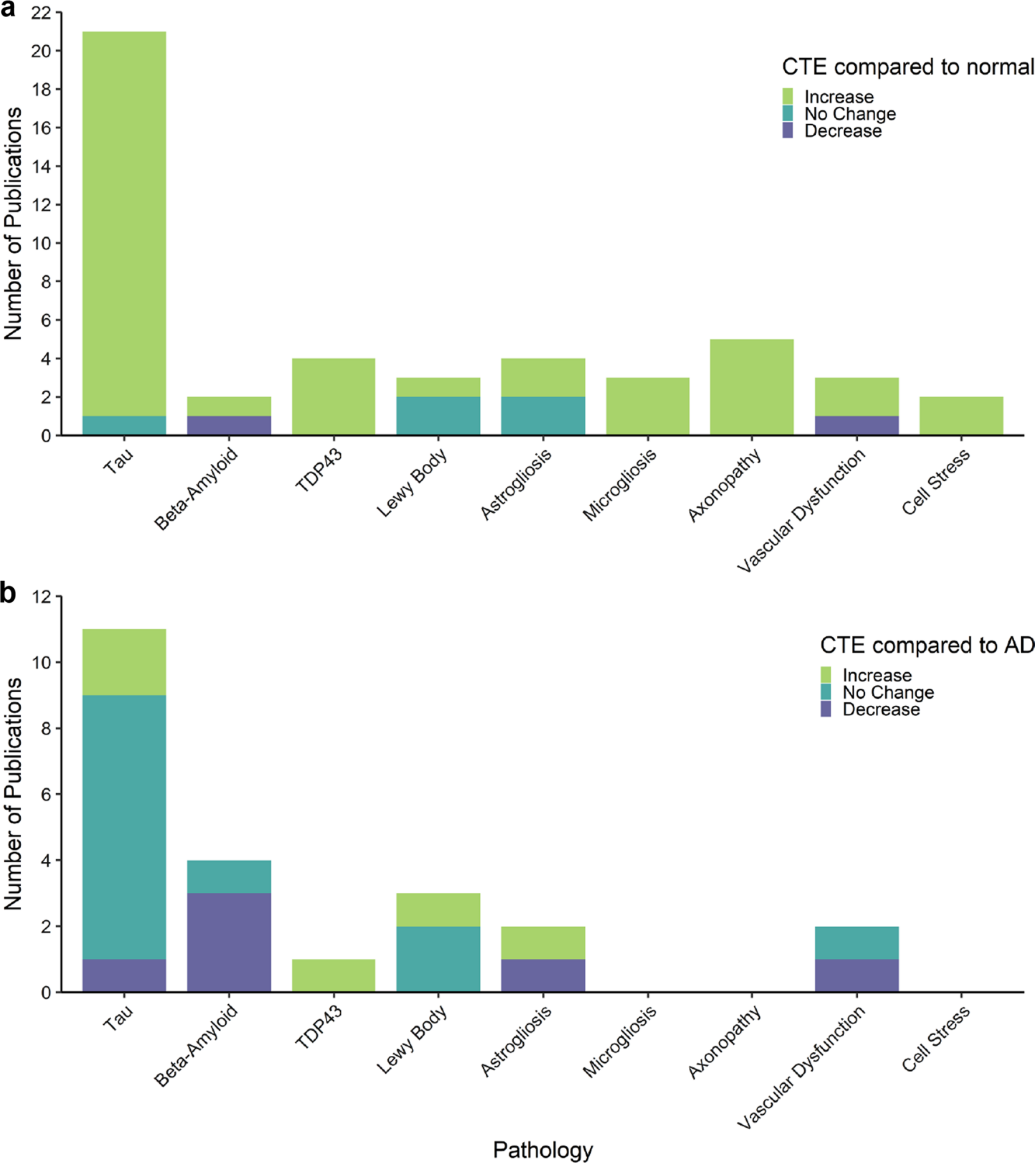
Summary of pathology findings in CTE postmortem tissue neuropathology studies. Stacked bar charts illustrating the number of human postmortem tissue publications that describe an increase, no change or decrease in CTE cases compared to neuropathologically normal cases (**a**) and Alzheimer’s disease cases (**b**) for each pathology category. Only studies where the amount of the pathology was compared between groups have been included in these graphs. Studies that describe differences in distribution were not included. Note that studies in each pathology category investigated different brain regions

For those studies comparing CTE to normal aged cases, the direction of the reported pathological change was generally consistent. All studies examining p-tau, TDP-43, microgliosis, axonopathy and cell stress reported an increase in the relevant markers in CTE cases compared to normal aged cases. Contradictory conclusions were presented in studies that examined beta-amyloid and vascular dysfunction in CTE compared to normal aging (Fig. 3a). The comparisons between CTE and AD cases were conflicting for most pathology categories, including p-tau, beta-amyloid, astrogliosis and vascular dysfunction (Fig. 3b). It is important to note that many studies had small sample sizes for the disease groups (Table 1) and groups were not age-matched in all studies (Additional file 1). We also noted that microgliosis, axonopathy and cell stress markers were not compared between CTE and AD in any of the 42 studies reviewed. A detailed description of the conclusions from each study for each pathology category is provided in the Additional file 1.

#### Tau

The CTE pathognomonic lesion is characterised by the perivascular accumulation of p-tau aggregates in neurons as pretangles, NFTs and thread-like neurites, either with or without thorn-shaped astrocytic p-tau, in the depths of the cortical sulci [16, 58]. Therefore, it is unsurprising that aggregated p-tau is the most frequently investigated pathology in comparative postmortem histology studies of CTE (31/42 publications reviewed). Increased p-tau load in CTE cases relative to normal aging is expected as the diagnostic criteria for CTE require the presence of perivascular p-tau pathology in the cortical sulci [58]. Accordingly, we found that 21 of the 31 p-tau studies compared the amount of p-tau between CTE and normal aged cases, and there was a clear consensus that p-tau pathology is increased in CTE. Increased p-tau NFT pathology has been reported in the frontal [2, 26–28, 30, 37, 48, 50, 78, 80], parietal [37, 48, 78, 80] and occipital cortices [48, 78], the temporal lobe [30, 36, 37, 46, 48, 61, 74, 76, 78] including the entorhinal cortex [48, 54] and hippocampal formation [48, 66], and other specific regions including the spinal cord [59, 66], locus coeruleus [55] and superior colliculus [11] of CTE cases compared to normal aged cases.

Fewer studies have compared the amount of p-tau between CTE and AD cases (11/31), and the majority report no difference in the overall amount of p-tau between these two groups [6, 7, 10, 12, 44, 46, 48, 75, 76]. Several studies highlight differences in the types of cells affected and the regional and laminar distribution of p-tau between CTE and AD [8, 13, 23, 40]. The publications that did report differences in the amount of p-tau between CTE and AD cases studied only five or fewer CTE cases. These publications include: (1) a report of increased p-tau NFTs in the locus coeruleus of CTE cases compared to AD [55]. (2) One study of the temporal lobe that reported an increase in labelling of the conformation-specific p-tau antibody Alz-50 in the gyrus of dementia pugilistica cases compared to AD [61]. (3) A study of the frontal and temporal lobe cortices that reported a decrease in the number of NFTs in CTE compared to AD [13]. In both CTE and AD, the amount of p-tau in each region increases at each progressive pathological stage [48] and many studies were published prior to the McKee staging criteria or did not report the CTE staging. Studies also often include cases from a range of CTE and AD stages, so a direct comparison of p-tau load across mixed stage cohorts is less informative. Therefore, rather than the absolute amount of p-tau differing between CTE and AD, current literature focuses on p-tau NFT composition and distribution.

The distribution of p-tau is emerging as one of the distinguishing features of CTE. Relative to AD, p-tau in CTE is primarily localised at the sulcal depths in early disease stages but becomes more uniformly distributed across the cortex at later stages [6, 10]. One study reported more astrocytic p-tau in the sulcal depths in CTE than in AD, with neuronal p-tau more evenly distributed between the gyral crest and sulcal depth in both diseases [6]. However, the 22 CTE cases used in this study had multiple comorbid pathologies and larger case series without comorbid pathology have reported neuronal p-tau to be concentrated in the sulcal depth rather than the gyral crest in CTE [6, 60]. The distribution of p-tau across cortical layers also appears to be distinct, with NFTs concentrating in the superficial cortical layers in CTE and in the deeper cortical layers in AD [8, 13, 40]. Furthermore, a detailed study of hippocampal NFT composition found hippocampal pathology can aid in differentiation of CTE and AD as the CA2/CA3 and CA4 subregions of the hippocampus are more severely affected than the CA1 region in CTE, while AD cases show more p-tau pathology in the subiculum and CA1 region [23]. Overall, there is consensus between studies that differences in p-tau distribution are features that help distinguish between CTE and AD pathology.

Differences in p-tau composition is increasingly recognised as a potential differentiating feature of CTE. A recent study that performed p-tau phenotyping on postmortem tissue from CTE, AD and other tauopathies showed that the immunoreactivity profile of different p-tau antibodies in CTE is similar to that of age-related tau pathology (PART/ARTAG) and AD [7]. Neuronal NFTs and astrocytic p-tau had a distinct phenotype that was conserved between CTE, PART, ARTAG and AD, where NFTs are composed of both 3R and 4R p-tau, while astrocytic p-tau was only immunoreactive for 4R p-tau antibodies [7]. This same finding was reported in a descriptive study of neuronal and glial p-tau isoforms in CTE (not included in this review) [24]. The authors propose that these phenotypic similarities between CTE, age-related p-tau pathology and AD suggest a common pathogenic mechanism [7]. However, differences in hippocampal and temporal lobe p-tau isoform composition have been noted between CTE and AD. At all stages of AD severity, NFTs containing 4R p-tau were more abundant than those containing 3R p-tau while in CTE cases, there was an equivalent amount of 3R and 4R NFTs [23]. Furthermore, the relative amount of different p-tau epitopes in CTE and AD frontal cortex were found to differ in a study of homogenised tissue (not included in this review due to absence of histology) [81]. This study found tau phosphorylated at serine 202 (p-tau_202_) was more abundant in CTE cases and significantly associated with increased years of head injury exposure, while tau phosphorylated at serine 396 (p-tau_396_) was most abundant in AD cases. The ratio of p-tau_202_: p-tau_396_ was increased in low and high severity CTE cases compared to AD [81]. Quantitative NFT phenotyping in different brain regions and using antibodies against different phosphorylation epitopes and tau isoforms may therefore reveal more subtle differences in p-tau pathology between diseases.

#### Beta-amyloid

Beta-amyloid pathology is a characteristic feature of AD but is also frequently observed in neuropathologically normal aged individuals. Extracellular beta-amyloid plaques can be classified as either diffuse or neuritic based on the plaque morphology. Neuritic plaques are compact argyrophilic aggregations of beta-amyloid that can also contain neurofibrillary tangles, dystrophic neurites, and a dense amyloid core. Diffuse plaques are less compact and lack a dense core or dystrophic neurites [32]. Neuritic plaques are significantly associated with cognitive deficits while diffuse plaques are common in the brain of neurologically normal elderly individuals [79]. The diagnosis of AD neuropathological change emphasises the presence of neuritic plaques across all six layers of the isocortex and it is acknowledged that sparse diffuse plaques may be observed throughout the cortex of neurologically normal elderly individuals [63, 64, 79]. Both diffuse and neuritic plaques have been reported in CTE cases [84]. Beta-amyloid pathology also includes cerebral amyloid angiopathy (CAA), where beta-amyloid is deposited in the walls of the brain vasculature. Beta-amyloid was the second most frequently investigated pathology in studies of CTE postmortem tissue (6/42 publications reviewed).

One of these studies exclusively examined the presence of CAA in CTE cases relative to normal aging and AD groups. They reported that the overall frequency of CAA in their CTE cohort (28.7%, 72/251 cases) was less than that of the normal aging group (54.3%, 153/282 cases) and AD group (95.7%, 220/230 cases), however, it is important to note that age is a strong confounding variable in this study given the CTE cohort studied was significantly younger than the normal ageing and AD cohort [80]. This study proposed that the distribution of CAA may be a distinguishing feature of CTE and AD. More prevalent and severe CAA was seen in the frontal leptomeningeal vessels than the intracortical vessels of CTE cases, while in AD cases, the parietal leptomeninges and intracortical vessels were more frequently and severely affected than those of the frontal lobe. As the frontal lobe is also the most frequent initial site of CTE p-tau lesions, future studies investigating the spatial relationship between CAA and p-tau perivascular lesions in CTE would help explain whether these pathologies are linked.

Of the other five studies of beta-amyloid pathology, only one compared the number of plaques between CTE and normal aging cases. Roberts et al. [76] report an increased number of beta-amyloid plaques in the dementia pugilistica cases from the Corsellis collection compared to normal aged cases [76]. Four studies reported that beta-amyloid plaque load was decreased in CTE compared to AD cases [8, 9, 40, 84], but also that the type and distribution of plaques differed. However, all four of these studies included CTE cases with a younger mean age than the AD cases and only one of the four studies had more than 10 CTE cases. A comprehensive assessment of beta-amyloid deposition in the dorsolateral frontal cortex by Stein et al. [84] reported diffuse and/or neuritic plaque pathology in 52% of their CTE cohort (n = 114) with a mean age of 60, suggesting an acceleration of beta-amyloid deposition in CTE compared to normal ageing [84]. They also found that AD cases had more neuritic plaques than CTE cases but a similar frequency of diffuse plaques. In addition, CTE cases with comorbid AD pathology showed a greater frequency of plaques labelled for Aβ1-40 at sulcal depths compared to the gyrus, while there was no difference in density of plaques labelled for Aβ1-42 between the sulcus and gyrus. This predominantly sulcal distribution of Aβ1-40 labelling was only seen in CTE cases with comorbid AD pathology and was not present in AD cases or CTE cases with only neuritic plaque pathology [84]. Armstrong et al. [9] also illustrate that beta-amyloid plaques are more evenly distributed throughout the cortical layers in CTE cases, while in AD cases, the superficial cortical layers are more severely affected [9]. Therefore, the distribution and type of plaque pathology appear to differ between diseases.

Beta-amyloid pathology is also associated with age. Stein et al. [84] show that the frequency of plaques in the dorsolateral frontal cortex increases with the CTE stage, age of symptom onset, and age at death. The CTE cases without plaque pathology in this study were younger on average (48.2 ± 2.6 years) than those CTE cases with diffuse plaque pathology (mean: 71.6 ± 1.4 years) and neuritic plaque pathology (73.6 ± 1.4) [84]. McKee et al. [60] also found that CTE cases with beta-amyloid deposition were significantly older than those without. Studies that compare plaque deposition in CTE and AD also examined CTE cases with a mean age of ~ 70 years [8, 9], which is similar to the average age of the cases in the Corsellis collection that Roberts et al. [76] compared to normal aged cases (range 57–91 years; mean: 67 years).

These studies suggest that beta-amyloid plaque pathology is a frequent but likely age-related comorbidity in CTE that may contribute to the disease progression. There is a clear consensus that beta-amyloid plaque morphology, distribution, and load differ between CTE and AD cases, however, additional research is required to understand whether beta-amyloid plaque pathology differs between CTE and normal aging.

#### TDP-43

The accumulation of TAR DNA-binding protein 43 (TDP-43) is a pathology observed in ALS and FTD, where TDP-43 inclusions are present in the spinal cord and cortex [18, 49, 59]. TDP-43 is also a frequent comorbid pathology in AD; although is often confined to the limbic region [49, 59, 64]. Abnormally phosphorylated TDP-43 inclusions within neurons and glia are commonly observed in CTE cases and are a supporting feature in the CTE diagnostic criteria [16, 59, 60]. This pathology is often distributed in a similar pattern to that found in frontotemporal dementia and ALS, with widespread involvement of the cortex and extension into the spinal cord in some cases [59]. However, despite TDP-43 inclusions being a common comorbid pathology in CTE, we identified only four studies that had compared TDP-43 pathology between CTE and normal aging or AD groups. We found a strong consensus that TDP-43 pathology is elevated and widespread in CTE compared to normal aging and AD cases. All four studies reported that TDP-43 aggregation is absent in normal aged cases but present in spinal cord, hippocampus, amygdala, frontal cortex, medial temporal lobe, and/or basal ganglia of CTE cases [5, 49, 59, 60]. One study showed that TDP-43 inclusions were more abundant and widespread in CTE than AD, with a distribution more closely resembling FTD with TDP-43 inclusions [49]. That is, TDP-43 inclusions were present in limbic system and neocortical regions of CTE cases but were limited to the limbic regions in AD cases. However, it should be noted that only three CTE cases were examined in this study [49]. Further exploration of the distribution and cell populations affected by TDP-43 inclusions relative to p-tau NFTs would be warranted to understand the relationship between these aggregate pathologies and how they differ between diseases.

#### Lewy bodies

The aggregation of alpha-synuclein into Lewy bodies is a hallmark pathology of Parkinson’s disease and Dementia with Lewy Bodies and is a common comorbid pathology in AD. Four studies investigated the presence of Lewy bodies in CTE. Two of these studies found no Lewy bodies in CTE, normal aging, or AD cases [48, 55], although one exclusively examined the locus coeruleus [55] and both studies had small sample sizes. Of the two studies that did observe Lewy body pathology in CTE cases, one study reported that 15/68 CTE cases examined had alpha-synuclein Lewy bodies, 13 of which had a comorbid diagnosis of AD, Lewy body disease, and/or Parkinson’s disease and all were significantly older than those without Lewy bodies. In addition, the Lewy body pathology was restricted to the olfactory bulb and medulla in the 2/15 CTE cases without a comorbid diagnosis [60]. The second study, however, investigated a larger CTE cohort and reported Lewy body disease in 53/139 CTE cases aged over 50 years old, 17 of which also presented comorbid AD pathology. This study also demonstrated an association between years of repetitive head injury and neocortical Lewy body disease in both the contact sport group and a community aging cohort, indicating an association between repetitive head injury and Lewy body pathology [2]. Of the CTE and AD cases that did contain Lewy bodies, the CTE cases had a higher frequency of brainstem Lewy body pathology than AD cases, of which the majority were amygdala predominant. In addition, CTE cases with concomitant AD and Lewy body pathology had a greater frequency of neocortical Lewy body disease [2]. These histology studies with larger CTE group sizes suggest that Lewy body pathology is a frequent comorbid feature of CTE and highlight the need for further investigation into the interaction between CTE and Lewy body pathology in larger autopsy cohorts.

#### Astrogliosis

Reactive astrogliosis refers to increased recruitment and morphological and functional remodelling of astrocytes in response to stimulus by injury or disease processes [33]. Glial fibrillary acidic protein (GFAP) is the most widely used marker of reactive astrogliosis in human tissue studies of neurodegeneration. GFAP labels astrocyte intermediate filaments and is used to investigate astrocyte numbers and reactive morphological changes. These morphological changes are heterogenous but include hypertrophy of the cell body and its processes. Increased number and reactive morphology of GFAP + astrocytes are robust observations in AD brain tissue [47, 87]. Our systematic review identified three studies that examined GFAP in CTE compared to normal aged cases and found no difference in the number of GFAP + cells or area of GFAP immunoreactivity [21, 26, 44]. One of these studies demonstrated increased CD44 expression in CTE, which supports an increased astrocytic neuroinflammatory response despite no increase in the overall number of astrocytes [21]. Astrocytes in CTE cases also appeared to have a more degenerated morphology with “beaded, puncta-like GFAP immunoreactivity” [44]. One study compared GFAP immunoreactivity between CTE, AD and FTD cases and showed the labelling was more diffuse and concentrated in the sulcal depths in CTE cases relative to AD and FTD [44].

In terms of functional remodelling, our review found one study that demonstrated increased expression of NQO1 in CTE compared to normal aging and AD. NQO1 is a redox regulatory protein that is upregulated in response to oxidative stress, and this study observed co-labelling of NQO1 with GFAP in the CTE cases suggesting an increased oxidative stress response in astrocytes [27].

Overall, there is currently limited literature on the extent of reactive astrogliosis in CTE compared to AD or normal aging. A recent consensus statement proposes that morphological changes indicated by GFAP labelling alone is not sufficient to define reactive astrogliosis and a combination of molecular markers should be used [33]. Considering perivascular astrocytic p-tau is a prominent feature of CTE lesions, quantitative investigation of a comprehensive battery of reactive astrogliosis markers should be a focus of future CTE postmortem tissue studies.

#### Microgliosis

Microglial responses to disease or injury include increased recruitment and alterations to morphology and function [53]. Pathological stimulation is associated with a morphological shift from a highly ramified structure to a hypertrophic or amoeboid structure. Iba1 is a marker of macrophages and microglia that is frequently used to identify these morphological changes [43]. Immunohistochemistry studies typically report alterations in morphology and increased immunoreactivity for markers such as CD68 and HLA-DR as evidence of microglial activation in neurodegenerative diseases [43].

Three studies identified by our systematic review examined microglial activation in the dorsolateral frontal cortex of CTE compared to normal aged cases. One quantitative study reported no difference in the overall number of Iba1 + cells but more hypertrophic morphology in CTE cases and an increased number of CD68 + cells [26]. Similarly, HLA-DR immunoreactivity was increased in the white matter beneath p-tau lesions compared to more distant regions [37]. Focal microgliosis was also reported in a recent study examining blood vessels central to the CTE lesions compared to distant vessels. Microglial recruitment was observed around lesion vessels in the CTE cases, including a subset of Iba1 + /TMEM119- infiltrating peripheral macrophages, which were not present around distant blood vessels or vessels in normal aged cases [25].

Overall, the limited literature on microgliosis in CTE indicates there is localised microglial activation around CTE lesions. However, the complexity and heterogeneity of microglial responses suggest that further investigation of additional markers is warranted. We did not find any study comparing microgliosis in CTE and AD cases. Microglial activation has been extensively studied in AD and found to concentrate around beta-amyloid plaques [43]. A comparison of microglial activation between CTE and AD will indicate whether the neuroinflammatory process differs between these different p-tauopathies. Comparison between CTE cases with or without comorbid aggregated protein pathologies would also provide context for the heterogeneity of microglial responses.

#### Axonopathy and dendritic alterations

Five studies examined axonal or dendritic structural defects in cortical regions of CTE cases compared to normal aged cases. Two studies examining phospho-neurofilament heavy protein reported immunoreactivity that indicated axonal injury and varicosities in CTE cases but not in normals [37, 60]. Reduced immunoreactivity of axonal markers βIII-tubulin and myelin-associated glycoprotein is observed in both grey and white matter of CTE cases compared to normal aging, which indicate axonal alterations in CTE [15]. A decrease in the number of Olig2 + oligodendrocytes in the dorsolateral frontal cortex of CTE cases compared to normal aging was also reported, although there was no difference in the number of PDGFRα + oligodendrocyte precursor cells [21]. Lastly, dendritic structure has been compared between CTE and normal aging using Golgi staining, which revealed an overall loss of dendrites and dendritic spines on neurons in the frontal and occipital poles of CTE cases [89]. We did not identify any studies that have compared axonal or dendritic pathology between CTE and AD cases.

#### Vascular dysfunction

Studies that examined pathological changes to the vasculature such as altered vessel structure and composition or vessel leakage were included in the vascular dysfunction category. While the pathognomonic CTE lesion is defined by the presence of perivascular p-tau in the sulcal depths, only three studies have compared aspects of vascular dysfunction between CTE, normal aging, and other disease groups. One of these studies is the previously described examination of CAA which described the differential distribution of CAA-affected vessels in CTE and AD [80]. The other two studies qualitatively describe vascular damage in CTE cases compared to normal aged cases, including increased perivascular haemorrhage detected by iron labelling [1], microvascular fragmentation, reduced vascular branching, and an increased amount of string vessels [19] (note: Buée et al. [19] studied two CTE cases). The severity of these vascular changes was described as similar between CTE and AD cases. Quantitative examination of microvascular structure in CTE, AD and normal aged cases is lacking in the neuropathology literature.

#### Cell stress

Our systematic review identified three studies that examined pathways related to endoplasmic reticulum (ER) stress and protein degradation. The unfolded protein response (UPR) consists of three parallel signalling pathways that are triggered by misfolded proteins and ER stress. One study examined the UPR and found increased immunoreactivity of phospho-eIF2α, XBP1, ATF6 and CHOP in the entorhinal cortex of CTE cases compared to a neuropathologically normal case, concluding that all three arms of the UPR are chronically activated in CTE cases [54]. However, only two CTE cases and one normal case were examined. This study also demonstrated co-labelling of UPR activation markers with p-tau, a finding that has also been reported in studies of AD postmortem tissue [41, 67, 68]. However, no study was found comparing UPR activation in CTE and AD cases.

Ubiquitination is a post-translational modification that targets a protein for degradation by the 26S proteasome [38]. Ubiquitination of p-tau is a well-reported feature of AD pathology, but our systematic review found that no study has compared the amount of ubiquitin labelling in CTE and AD postmortem tissue. One study reported ubiquitin-positive NFTs in the frontal and temporal lobe of CTE cases at a higher frequency than in normal aged cases [30].

Proteins involved in nuclear pore complex and nucleocytoplasmic transport contribute to the regulation of protein synthesis. Disruptions to the nuclear pore complex have been reported in other neurodegenerative diseases, but only one study investigated this in CTE postmortem tissue [5]. Nuclear pore glycoprotein p62 (NUP62) is an essential component of the nuclear pore complex and immunoreactivity for NUP62 was found to be increased in CTE tissue compared to normal age-matched cases. Increased cytoplasmic NUP62 co-labelling with phospho-TDP-43 in severe CTE cases compared to mild cases suggests alterations to the nuclear pore complex are related to protein aggregation and disease severity [5].

## Discussion

This systematic review summarises the current landscape of comparative CTE histology literature. Our exploration shows a growing interest in CTE research based on the increasing number of neuropathology studies published per year. The increased rate of publications since 2015 reflects the uptake of the pathological diagnosis consensus criteria and the organised international effort to grow CTE brain banking operations. Overall, our systematic review focused on comparative histology studies to provide an overview of the pathologies that have been explored relative to normal aging or other disease groups. However, descriptive studies and studies utilising human tissue techniques other than histology that have been conducted to date also provide important insights that corroborate the findings of comparative histology studies.

### The distribution and composition of p-tau and other aggregate pathologies may be distinguishing features of CTE

Our review concluded that p-tau is the most studied pathology in CTE cases with limited investigations on other aggregate and non-aggregate pathologies. This is not surprising given the importance of p-tau as a diagnostic marker of CTE. In this review, the differences in composition and distribution of p-tau and the types of cells affected were identified as promising features for distinguishing CTE and other tauopathies as opposed to total p-tau pathology measures. Those differences in neuronal and astrocytic p-tau distribution and composition were highlighted as useful diagnostic features of CTE. Data supporting this concept was recently published in a study that fell outside the date range of this review [4]. This study showed that antibody labelling for p-tau isoforms present in both neuronal and astrocytic p-tau facilitates better detection of CTE pathology than antibodies that exclusively label neuronal p-tau. A descriptive study not included in this review also provides evidence that the development of astrocytic p-tau within the perivascular lesions increases with CTE disease severity [24]. Therefore, exploring the processes associated with p-tau aggregation in different cell populations may help explain the features of CTE progression.

Relative to p-tau pathology, beta-amyloid was studied in CTE cases to less extent. Based on the literature reviewed here, beta-amyloid is likely an age-related comorbidity that can occur alongside CTE p-tau pathology and contribute to disease progression. However, surprisingly little research compares plaque pathology in CTE and normal aging. While there are reported differences in plaque morphology, distribution, and load between CTE and AD cases, we have limited objective evidence of how plaque composition and distribution in CTE differs from that seen in age-matched neuropathologically normal individuals. Stein et al. [84] provides the most comprehensive assessment of plaque pathology in CTE to date, showing that CTE cases with beta-amyloid plaques have more severe p-tau pathology, a more aggressive clinical syndrome, and more frequent comorbid Lewy body pathology. Beta-amyloid deposition has also been linked to axonal injury, which specifically occurs at the sulci of CTE cases [84]. Future studies that compare plaque composition in the cortical sulci and gyrus in both CTE and normal aged individuals are warranted, considering the potential role of plaque pathology in disease progression.

Overall, the scope of current comparative literature on comorbid aggregate pathologies in CTE compared to other neurodegenerative diseases is limited. The spatial relationship between p-tau NFTs, beta-amyloid plaques, TDP-43 inclusions and alpha-synuclein Lewy bodies is unclear. Furthermore, how these aggregate pathologies relate to other processes such as microgliosis, astrogliosis, vascular damage, and white matter damage is yet to be understood. Further studies into these relationships and how they differ between disease groups could provide valuable insights into the underlying processes that link head injury and degeneration in many diseases.

### Non-aggregate pathologies in CTE require further investigation

A key conclusion from this review is that the comparative literature examining non-aggregate pathologies in CTE is minimal. Pathologies such as vascular dysfunction, axonal damage and neuroinflammation occur acutely after head injury and persist, implicating them as possible initiating mechanisms of CTE. The spatial distribution of these pathologies is particularly relevant as the forces caused by head impact are concentrated in regions of structural inhomogeneity such as the sulcal depth and the vessel-tissue boundary [22]. Anatomical studies of non-aggregate pathologies are therefore key to understanding the disease process in CTE.

It is particularly striking to note that although the perivascular accumulation of p-tau is a defining feature of CTE, there has been no detailed quantitative analysis of blood vessel integrity. The qualitative studies indicate that CTE cases show features of vascular dysfunction relative to normal aged cases. A descriptive study by Alosco et al. [3] supports this by demonstrating moderate to severe arteriolosclerosis in 47.2% of the 180 CTE cases examined [3]. More research examining the relationship between vascular pathology, CAA, perivascular p-tau lesions, and neuroinflammation should be a priority for the field.

Neuroinflammation also has important links to brain vasculature and head injury. While it is a complex process that is difficult to study in postmortem tissue, the inflammatory process may be a key link between acute head injury and chronic degeneration and further investigations are warranted. Key examples of inflammatory involvement in CTE include astrogliosis and microglial activation.

Astrogliosis is a growing area of interest in CTE pathology, however, detailed anatomical studies profiling the extent and distribution of astrocyte activation are not yet available. We found that current studies of astrogliosis in CTE, AD and normal ageing predominantly use the marker GFAP, and we propose that future work should focus on a broader range of markers to characterise the heterogeneity of astrocyte responses in CTE. Single-nucleus RNA sequencing by Chancellor et al. [21] found a range of differentially expressed genes in the CTE astrocyte samples compared to normals that would warrant histological analysis and comparison to AD. Astrogliosis could contribute to progression from acute head injury to chronic degeneration as astrocyte endfeet are a component of the neurovascular unit and play an essential role in regulating glymphatic clearance of proteins like p-tau and beta-amyloid through the expression of the water channel aquaporin IV. However, when astrocytes become reactive, the perivascular presence of aquaporin IV is lost, which can lead to impaired glymphatic clearance and accumulation of p-tau and beta-amyloid [22, 45]. Therefore, future histology studies investigating the spatial relationship between markers of astrogliosis, aggregated protein pathologies and vasculature would be valuable.

Microglial activation is another aspect of neuroinflammation that has received recent attention. The spectrum of microglial responses is complex, but the studies reviewed here indicate that markers indicative of activation are increased relative to CTE disease severity, supporting the conclusion that neuroinflammation is an important disease process. A recent detailed study by Cherry et al. examining microglia around lesion and non-lesion vessels highlights the focal neuroinflammatory effects in CTE [25]. Efforts to compare this pattern of neuroinflammation between CTE and other diseases could help explain this central pathogenic process.

White matter pathology is also a key tissue feature that should be examined further in CTE. Specifically, the involvement of axonal damage in disease pathogenesis. Our systematic review found limited histological investigations into axonal damage and CTE; however, all studies investigating axonopathy demonstrated it was a consistent neuropathological feature of the disease. Furthermore, this pathology was shown to increase with increasing severity of disease and in concert with other pathological features such as p-tau and TDP-43. The specific mechanisms behind this pathology are not yet known in CTE. However, there is likely a relationship between acceleration/deceleration forces during head impacts causing the shearing of axons [17] and further degeneration influenced by p-tau and TDP-43 accumulation. Finally, cell stress features have been highlighted as a key contributor to neurodegenerative diseases but are yet to be thoroughly examined in CTE. An example is the contribution of the UPR to disease pathogenesis. Chronic UPR activation has been linked to synaptic failure and neuronal loss and is seen in other neurodegenerative diseases such as AD and PD [65]. Furthermore, in postmortem tissue studies of AD, UPR activation is evident in the hippocampus, frontal cortex, temporal cortex, and olfactory bulb within neurons that contain diffuse p-tau aggregates leading to the hypothesis that UPR activation precedes p-tau formation [41, 42, 67, 85]. In this review only one study investigated activation of the UPR and showed activation of all three arms of the UPR as well as co-labelling with p-tau pathology in CTE. In addition, UPR activation has also been shown to occur in response to TBI in rodent models, supporting the hypothesis that UPR activation may be one of the pathogenic mechanisms linking acute brain injury and neurodegeneration [14, 51, 54]. In summary, we propose that the investigation of non-aggregate pathologies should be a priority for future histology studies to improve our understanding of the CTE disease process.

### Limitations of tissue studies and review methodology

A limitation of the current literature is the considerble lack of comparative studies of CTE neuropathology. This is especially noticeable when considering pathologies outside of p-tau. While CTE research is still an emerging field, to better understand specific CTE pathology and the complex relationships between these pathologies, future research needs to shift from focusing on descriptive case studies to more comparative studies that include neuropathologically normal and neurological disease cases. However, it is important to acknowledge that this systematic review only examined histology studies and excluded studies that assessed pathological features of human brain tissue with other techniques such as western blot or ELISA. This exclusion results in some pathological features being underrepresented in our review, however it does highlight the need for additional histology investigations in these areas.

There is some argument in the literature about whether there is a distinction between “modern” CTE and “classic” CTE, where classic CTE refers to the early studies that describe dementia pugilistica in boxers [35]. This argument essentially questions whether earlier studies investigating dementia pugilistica were sufficiently detailed for the cases to be considered as “modern” CTE, given the different methods of identifying and describing pathologies compared to the criteria used today [57]. At this stage, there appears to be limited evidence supporting a distinction between the two diseases, and as such, the terms ‘dementia pugilistica’ and ‘CTE’ are widely considered to represent the same disease [20, 39, 57]. Therefore, both terms were included when searching for literature in this review.

In addition, throughout this review, we found discrepancies in how CTE and AD stage and severity were reported, and the brain region examined in each study. As pathology progressively affects different brain regions in both CTE and AD, to compare findings across studies effectively it is necessary to know the pathology stage of the cases. Revised criteria for defining CTE severity as ‘low’ or ‘high’ was recently published and will help overcome this issue in future studies [16].

Similarly, in this review, we summarised the overall change in the amount of pathology between groups reported by each study based on the amount of labelling described or quantified. The intent of this summary is to assess the relative consensus of studies in each pathology category; however, we acknowledge that this is a simplistic categorisation of complex findings given the different sample sizes and brain regions investigated between studies. It is evident that pathological changes in CTE may not differ in the amount of pathology but rather by its distribution and composition. This is particularly noticeable for p-tau pathology; however, it is also a likely feature of non-aggregate pathologies such as inflammation and vascular dysfunction and may help distinguish CTE from other disease groups. Therefore, studies that only assessed pathology distribution are not included in our summary analysis. Our overall assessment of the literature suggests that future studies should investigate the distribution and composition of non-aggregate pathologies and the relative amount between groups.

Limitations of neuropathological studies must also be considered. Comparative studies comprised a mixture of qualitative, semi-quantitative, and quantitative analysis methods. The inherent bias in qualitative immunohistochemistry studies is the tendency to report increased labelling, which is typically more apparent than reduced immunoreactivity [87]. There are also differences in the postmortem delay, fixation type and duration, and sectioning of tissue across studies. In terms of methodology, reporting of antigen retrieval techniques and specific antibody information was not consistent between studies. In addition, due to the relatively small number of studies that met the criteria for this review and the high variation in how methods were reported, we did not impose any quality threshold on the included studies. Therefore, several studies included in this review had small sample sizes or the disease groups were not appropriately age-matched, limiting the certainty in the findings. In the results section we have specifically noted studies that investigated less than five CTE cases and where there were large differences in age between cohorts. Despite these limitations, this systematic review provides a valuable summary of the histology literature that examines the neuropathology of CTE relative to aging and AD. Our findings highlight areas of clear consensus and areas that warrant further investigation.

### More studies of CTE tissue from individuals with diverse head injury exposure profiles are needed

A key finding raised in this review is that most studies in current literature use tissue acquired by the VA-BU-CLF brain bank. While this is intuitive, as the VA-BU-CLF brain bank houses the most extensive collection of brains from individuals exposed to significant repetitive head injury, it shows that current CTE pathology literature is largely based on extensive examination of a specific brain bank cohort. From our analysis of head injury exposure, we found that CTE neuropathology has been studied in athletes from a range of contact sports; however, 60% of these publications studied the brains of former American football athletes, and of these studies, 84% were acquired from the VA-BU-CLF brain bank. It is important to note that we were not able to determine whether specific CTE cases were used for more than one study as this information was not reported. Therefore, we cannot assess the breadth of cases used in current literature. The number of CTE cases currently diagnosed globally was recently summarised in a review by Nowinski et al. [69].

Our analysis also showed that current postmortem histology studies exclusively study CTE tissue from male donors. This reflects the demographics of contact sport participation, particularly American football, the most common primary head injury exposure of donors to the VA-BU-CLF brain bank. The increased number of women in contact-sport careers is a recent development, and future studies will need to investigate potential sex-related differences in CTE pathology as the tissue becomes available.

The issue of selection bias in CTE brain donation and research studies is frequently acknowledged in the publications we assessed, and at this early stage of research where global brain banking efforts are still developing these issues of case selection are unavoidable. There is value in studying homogenous populations to identify CTE-specific pathologies by limiting confounding variables. These disease-specific features will provide insights into the mechanisms that drive CTE pathology. Comparative studies of CTE pathology in individuals who died very young and did not have age-related brain changes could also help identify pathology signatures other than p-tau that can contribute to an antemortem diagnosis. Such signatures could also improve the postmortem classification of CTE as the focal p-tau lesions can be sparse or masked by complex comorbid pathologies in advanced stages or in elderly individuals. However, it is important that we also study CTE in individuals with more diverse demographics and sources of head injury exposure to ensure pathological findings are widely applicable and to further understand the potential heterogeneity of CTE pathology.

The expanding diversity of CTE brain tissue will also inevitably present challenges for comparative studies. This review highlights that comorbid aggregate pathologies are common in CTE, with TDP-43 inclusions, Lewy bodies, beta-amyloid plaques, and CAA frequently observed alongside the characteristic p-tau lesions. This complex aggregate pathology aligns with the extensive evidence implicating traumatic brain injury as a risk factor for many neurodegenerative diseases [34, 73, 90, 91]. However, the occurrence of multiple pathologies also increases with age, and the studies reviewed here indicate that is also true for CTE cases with an older age at death. The development of comorbid pathology is likely related to age, the severity and amount of exposure to repetitive head injury, and other individual genetic and environmental factors [83]. With increased global brain banking efforts, there will be an increased frequency of CTE cases with an older age at death and comorbid pathology. While comorbid pathology makes it difficult to group cases for comparative studies, it is important that future CTE research explores the complex relationships between aggregate pathologies where possible. Efforts to account for other aggregate pathologies and investigate their spatial relationships will provide insights relevant to all neurodegenerative diseases.

## Conclusion

In conclusion, current literature suggests that the distribution and composition of p-tau and other comorbid aggregate pathologies are the most distinguishing features of CTE. However, understanding the scope of other non-aggregate pathologies is key to understanding the disease mechanism and the relationship between CTE and other neurodegenerative diseases, for which head injury is also a risk factor. This systematic review has highlighted that: (1) pathologies such as vascular dysfunction, gliosis, axonopathy, and cell stress require further and focused investigation. (2) More studies of CTE tissue from individuals with diverse head injury exposure profiles are needed to understand the heterogeneity of CTE pathology. (3) Future neuropathology research should focus on detailed comparisons of CTE, normal aging, and other neurodegenerative diseases. Together with the growing interest in CTE research and international efforts to grow CTE brain banking initiatives, we believe that these priorities will help accelerate our understanding of this disease.

## Supplementary Information

**Additional file 1.** Summary of data acquired from all studies examined in this review.

## Data Availability

All data reported in this systematic review was obtained from the articles. The data used for this study is available in the supplementary files. An interactive visualisation of the data can be found at: https://public.tableau.com/app/profile/helen.murray4711/viz/MorethanaKnocktotheHead/Morethanaknocktothehead.

## Abbreviations

AD: Alzheimer’s disease
ALS: Amyotrophic lateral sclerosis
ARTAG: Aging-related tau astrogliopathy
CAA: Cerebral amyloid angiopathy
CTE: Chronic traumatic encephalopathy
ER: Endoplasmic reticulum
NFT: Neurofibrillary tangles
NUP-62: Nucleoporin 62
PART: Primary age-related tauopathy
TDP-43: TAR DNA binding protein 43
UNITE: Understanding Neurological Injury and Traumatic Encephalopathy
UPR: Unfolded protein response
VA-BU-CLF: Veteran Affairs-Boston University-Concussion Legacy Foundation
